# Modulation of the blood-tumor barrier to enhance drug delivery and efficacy for brain metastases

**DOI:** 10.1093/noajnl/vdab123

**Published:** 2021-11-27

**Authors:** Kathryn E Blethen, Tasneem A Arsiwala, Ross A Fladeland, Samuel A Sprowls, Dhruvi M Panchal, Chris E Adkins, Brooke N Kielkowski, Leland E Earp, Morgan J Glass, Trenton A Pritt, Yssabela M Cabuyao, Sonikpreet Aulakh, Paul R Lockman

**Affiliations:** 1 Department of Basic Pharmaceutical Sciences, School of Pharmacy, West Virginia University, Morgantown, West Virginia, USA; 2 Department of Chemical and Biomedical Engineering, Benjamin M. Statler College of Engineering and Mineral Resources, West Virginia University, Morgantown, West Virginia, USA; 3 Department of Cancer Cell Biology, School of Medicine, West Virginia University, Morgantown, West Virginia, USA; 4 Department of Pharmaceutical Sciences, School of Pharmacy, South University, Savannah, Georgia, USA

**Keywords:** blood-brain barrier, brain metastases, breast cancer, drug delivery

## Abstract

The blood-brain barrier is the selectively permeable vasculature of the brain vital for maintaining homeostasis and neurological function. Low permeability is beneficial in the presence of toxins and pathogens in the blood. However, in the presence of metastatic brain tumors, it is a challenge for drug delivery. Although the blood-tumor barrier is slightly leaky, it still is not permissive enough to allow the accumulation of therapeutic drug concentrations in brain metastases. Herein, we discuss the differences between primary brain tumors and metastatic brain tumors vasculature, effects of therapeutics on the blood-tumor barrier, and characteristics to be manipulated for more effective drug delivery.

## Blood-Brain Barrier (BBB) and Blood-Tumor Barrier (BTB)

The BBB is comprised of a neurovascular unit (NVU) consisting of capillary endothelial cells, pericytes, astrocytes, and a basement membrane.^1^ The innermost layer of the BBB is formed by endothelial cells which establish a barrier between circulating blood and the brain parenchyma.^2^ A basement membrane of extracellular matrix and pericytes envelops the endothelial cells to support structural integrity.^3^ Astrocytic end feet are located along the outermost layer of the NVU and play a significant role in regulatory processes such as K^+^ buffering, brain pH, and other metabolic processes.^4^ Interactions between these cells and their microenvironment are vital to maintain BBB integrity and brain homeostasis. However, when cancer cells displace endothelia from the other NVU cells the BBB breaks down and solute movement whether passive or actively transported is altered. It is important to note most of what is known about the BBB and BTB is due to preclinical work in mouse models.

### Brain Metastases vs Glioblastoma

The BTB restricts chemotherapeutic efficacy and contributes to tumor progression in both primary and metastatic brain tumors. Glioblastoma (GBM) is the most common, malignant primary brain tumor characterized often by a hypoxic necrotic center and invasive growth into normal brain tissue.^5^ Disruption of the BBB by invasive GBM was long considered uniformly leaky but is now understood to have a nonuniform, heterogeneous microvasculature composition with increasing distance from the tumor core.^6^ In early states, that is, low-grade glioma, the BBB remains nominally intact and little disruption is present as the tumor relies on the normal brain microvasculature.^7^ However, this changes dramatically as the tumor grows and progresses into a higher grade glioma where tumor cells, through a variety of molecular signals, drive the separation of endothelial tight junctions, dissociation of astrocytic processes, and recruitment of differential pericyte populations.^8^ Malignant GBM cells are also highly migratory and remodel the extravascular basement membrane through release of several soluble factors and induction of a cascade of pro-tumorigenic pathways.^4,9,10^ These properties help promote both chemotherapeutic and radiation therapy resistance as the leading edge of the tumor continues to expand and co-opt existing brain capillaries.^7^

**Figure 1.**
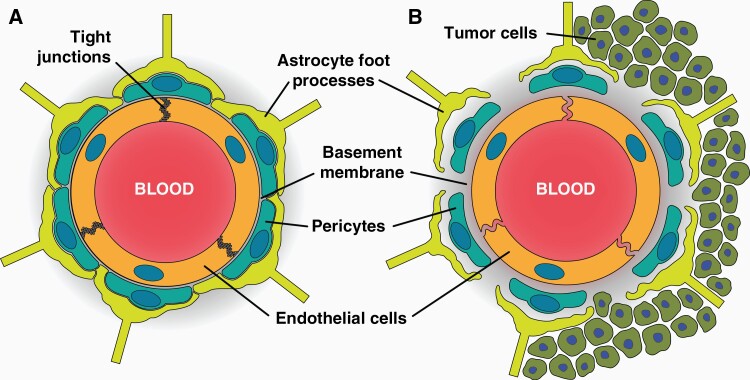
Differences between the BBB and BTB. (A) Endothelial cells in a healthy brain are held together by tight junction proteins and prevent paracellular transport. The endothelial cells are surrounded by a basement membrane embedded with pericytes and astrocytic foot processes along the outside. (B) The BTB is leakier than the BBB due to lack of tight junction proteins and decreased association of astrocytic end-foot processes and pericytes with the barrier.

In contrast to the development of GBM, metastatic brain tumors arise from a peripheral primary tumor location. The most common cancer types contributing to the formation of brain metastases are lung, breast, and melanoma.^11^ Initial steps of lesion formation are similar to immune cell trans-endothelial migration, which include tethering, rolling, adhesion, and diapedesis.^12^ Extravasating into the brain parenchyma beyond the endothelia level has been observed to take longer than in other organs ranging between 2 and 14 days depending upon primary tumor type.^13,14^ As the metastatic cells continue to grow beyond the BBB, nutrient and oxygen demand increase leading to vascular co-option, a process by which tumor cells alter the existing brain microvasculature.^15^ Simultaneously, angiogenesis occurs to provide cancer cells with nutrients to support proliferation and survival. The immature vessels formed during this process are fenestrated and lack endothelial tight junction protein complexes allowing increased vascular permeability.^16^ These immature capillaries are “leaky” compared to normal BBB capillaries.^17^ While the lesion continues to grow in size, the tumor becomes more hypoxic and secretes vascular endothelial growth factor (VEGF) to induce more angiogenesis.^18^ This dynamic process contributes to BTB permeability. Interestingly, no correlation has been observed between lesion size and vascular permeability in preclinical brain metastasis models.^17,19,20^ Clinically, substantial intra- and inter-tumoral heterogeneity exists among brain metastases in the same brain.^17,21,22^ Differences between the BBB and BTB are shown in Figure 1.

### Heterogeneity of Blood-Tumor Barrier in Brain Metastases

There are relatively few studies comparing the vascular permeability of metastatic brain lesions (regardless of type) to a primary tumor. The data presented are often difficult to reconcile due to varying methods clinically available and disparities in how a given method is conducted. For example, in comparing data between CT perfusion studies for astrocytomas and GBM vs central metastatic lesions from lung, breast, and melanoma the permeability values (rPSmax) for primary tumors are largely 10 times the values of metastasis lesions in the brain. Though it should be noted astrocytoma values fell more in line with metastatic lesions compared to the primary tumors.^23,24^ While these data generally agree with preclinical data using Ktrans, calculations obtained with MRI show metastases have approximately 2/3rd of primary tumor permeability measurements.^25^ However, these data were a compilation of several lesion types, which could be the driving factor for this difference.

The BTB is anatomically different in brain metastases of breast cancer compared to a primary CNS tumor, which may help mechanistically explain variability and differences in permeability. The density of blood vessels in brain metastases of breast cancer lesions in mice is 40%-80% less than the vascular density of normal brain. Further, and more critical to this discussion the vascular density of the metastatic lesion is often only 12%-15% of a GBM.^26^ Vascular density alone suggests permeability would be reduced in the lesion compared to the primary tumor. However, the defects in the respective BTB vasculature are also indicative. In preclinical glioma models, the size of the vascular defect (pore; cylindrical opening through the endothelial wall) is at a minimum of ~150 nm in size. This opening is large enough for antibodies to freely penetrate from the blood to the tumor. However, the pore size within metastases in the brain is roughly ~5-9 nm in diameter, though there is likely variability of actual pore sizes in the vasculature within and between the various lesions in the same brain.^27^ The data suggest while the metastatic lesion will enhance with MRI, trastuzumab at an approximate size of 5.5-6 nm will be restricted from diffusing across vascular pores into the metastatic lesion to the degree it would have a clinical effect.

Our preclinical work generally agrees the BTB in metastatic lesions have permeability values less than a primary tumor, but also have some degree of compromise regardless of the lesions size, location, and/or tumor type.^20,27–34^ Though, there is subtly in this assertion. The vascular permeability in brain metastases can range from 1.1- to 100-fold, depending on the polarity and size of the marker. For example, the small (104 Da) charged zwitterion amino-isobutyric acid (AIB) penetrates lesions from 1.1-fold to upwards of 35-fold higher compared to the normal brain vasculature. The marker AIB is a small water-soluble marker that should easily penetrate defects induced in the vasculature within a metastatic lesion. However, water-soluble molecules such as antibodies (~150 kDa) have significantly less permeability through the BTB defects (~1.01- to ~3-fold; compared to normal brain).

Clinically, there are reports of a subset of breast cancer brain metastases that poorly or do not enhance with MRI, yet a large majority do. For the lesions that enhance, it may not be uniform throughout the lesion, leaving detection of total tumor mass difficult at times.^35^ Our preclinical data strongly agree with the heterogeneity of permeability seen within a lesion and between lesions in the same brain. When evaluating the distribution of lesion permeability of a small molecular weight marker the majority of lesions (~80%) had permeability increases of 1.5- to 3-fold and only 10% of lesions had permeation increases of greater than 10 compared to normal brain.^17^ A very similar pattern of variability is seen when looking at permeability within a single lesion. We have observed permeability variances can range as much as 1.1- to 25-fold.^17^

There have been reports in the literature regarding the positive correlation between increasing size^36^ and nuclear compactness^37^ with permeability increases. However, we have evaluated thousands of lesions across multiple sized markers, and different preclinical brain metastases models and 80% of the lesions fail to significantly associate size and increases in permeability,^17^ which agrees with other data obtained with MRI.^35^

We have observed quantifiable permeability increases, albeit sometimes subtle, in nearly every metastatic brain lesion. It should be noted for us to quantify the slight permeability increases, we use quantitative multimodal fluorescent and laser phosphorescent autoradiography to detect spatial permeability changes at a 1-micron resolution and drug tissue concentrations of 1 femtogram.^38^ This technique was adapted from prior double or triple autoradiography techniques^39^ and is well suited to study preclinical metastases since it has <1-µm resolution (29) and with ^14^C-phosphorescence, tracer distribution can be mapped in 10-µm pixels at levels (~0.3 nCi/g). The variability in permeability has significant implications for the effective delivery of chemotherapeutics within and between lesions in the same brain.

Despite the breakdown of the BBB in brain metastases of breast cancer, it still significantly restricts drug delivery and inhibits cytotoxicity in ~90% of CNS metastases.^20,27,34^ The poor delivery of chemotherapy within the brain lesion provides a sanctuary for the lesion to progress, in the presence of sub-therapeutic chemotherapy concentrations, within the brain microenvironment. Many chemotherapeutics exhibit restricted distribution because permeability increases are inadequate and or they are removed by efflux transporters that remain highly active despite the breakdown of the vasculature.^22,28^ This phenomenon we have observed for paclitaxel and doxorubicin^40,41^ as well as trastuzumab, lapatinib, and vorinostat.^38,42,43^

### Drug Delivery for Brain Metastases

Although many successful compounds have shown effectiveness in treating peripheral tumors with targeted agents, the same cannot be said for treating brain tumors. This lack of success may be due to inadequate delivery of otherwise effective compounds. Many factors affect how these drugs are delivered to the brain, but one major challenge is a heterogeneously leaky BTB. Future success of brain cancer therapeutics depends on the delivery of active drugs to the target at efficacious concentrations, which may include combinations of targeted drugs tailored to each patient’s tumor type.^44^

Traditional cytotoxic therapeutics have played a limited role in the treatment of brain metastases. The distribution of systemically administered chemotherapies is hampered by the BTB, which is frequently disrupted in patients with brain metastases.^45,46^ We analyzed over 2000 brain metastases in different preclinical models of metastatic breast cancer (human 231-BR-HER2 and murine 4T1-BR5) and found in over 89% of lesions, there was a partial compromise in BTB permeability. Nevertheless, the concentration of drugs only reached lethal levels in a small fraction (10%) of the most permeable metastases.^17^ Several trials evaluating the use of systemic drugs in patients with brain metastases have failed to demonstrate notable response rates, including cisplatin and pemetrexed,^47,48^ cisplatin and vinorelbine,^49^ paclitaxel and cisplatin,^50^ and temozolomide.^51,52^

Targeted therapies utilized to treat brain metastases have minimal brain distribution. Only 5% administered dose of Trastuzumab, which targets HER2^+^ breast cancer, is found in brain lesions in preclinical mouse models.^34^ Clinically, the ratio of trastuzumab in serum to cerebrospinal fluid is 420:1.^53^ Often a factor limiting drug delivery to brain metastases is the high degree of expression of ABC efflux transporters at the BBB and BTB. In preclinical studies, there is strong evidence for the interaction of vemurafenib, dabrafenib, trametinib, palbociclib, cobimetinib, doxorubicin, and paclitaxel being effluxed by P-gp (P-glycoprotein) and BCRP (breast cancer resistance protein). The brain concentrations achieved by most of these drugs are less than 10% of their plasma concentrations (Table 1). Moreover, the multidrug resistance protein 1 (MRP1) receptor acts by promoting drug resistance^54^ along with active efflux of drugs. This provides an additional challenge to achieving optimal drug concentrations across the BTB.^55^

**Table 1.** Brain-to-Plasma Ratio of Various P-gp and BCRP Substrate Chemotherapeutic Agents for the Treatment of Brain Metastases

Chemotherapeutic Agent	Cancer Type	Molecular Target	Clinical Status	Substrate		Brain-to-Plasma Ratio	Reference
				P-gp	BCRP		
Vemurafenib	Melanoma	Mutant BRAF	Approved	Yes	Yes	1.00 ± 0.19	112, 113
Dabrafenib	Melanoma, non–small-cell lung cancer, and thyroid cancer	Mutant BRAF	Approved	Yes	Yes	0.25	114
Trametinib	Melanoma, non–small-cell lung cancer, and thyroid cancer	MEK	Approved	Yes	No	2.45 ± 1.3	115
Cobimetinib	Melanoma	MEK	Approved	Yes	No	1.1, 6.2	116
Palbociclib	Breast cancer	CDK4/6	Approved	Yes	Yes	28 ± 6	117
Doxorubicin	Breast cancer, leukemia, lymphoma, ovarian cancer, neuroblastoma, bone cancer, and thyroid cancer	Topoisomerase II	Approved	Yes	Yes	0.0014	118
Paclitaxel	Breast cancer, lung cancer, ovarian cancer, and Kaposi’s sarcoma	Tubulin beta-1 chain, apoptosis regulator Bcl-2	Approved	Yes	No	<3%	^17,106^

### Immunotherapy for Brain Metastases

Although the CNS was once considered immune-privileged, studies have shown immune cells, specifically T cells, cross the BBB to perform immune surveillance.^56^ Brain metastases are now being researched as possible targets for a variety of immunotherapies, such as checkpoint inhibitors and adoptive cell therapy (ACT). The benefit of immunotherapy is immunosuppression without the toxicity associated with chemotherapeutic agents. However, the microenvironment of solid tumors can evade immune responses by impeding the infiltration of immune cells into the tumor, contributing to the variability in responses seen among patients.^57^

Checkpoint protein receptors, such as CTLA-4 (cytotoxic T-lymphocyte-associated antigen 4) and PD-1 (programmed death 1), are expressed on T cells. These checkpoints block immune responses and allow the tumor cells to evade the immune system. When checkpoints are inhibited, T cells are activated by the primary tumor then kill the cancer cells. Examples of immune checkpoint inhibitors are ipilimumab, which blocks CTLA-4, and pembrolizumab and nivolumab, which block the ligand PD-L1. Checkpoint immunotherapy is in clinical trials for patients with brain tumors, including advanced metastases and GBM. A 2010 study of individuals initially treated for metastatic melanoma which allowed enrollment of patients with treated CNS metastases was the first study to establish ipilimumab treatment improved survival.^58^ In addition, pembrolizumab and nivolumab have been clinically assessed for efficacy in patients with melanoma and lung cancer brain metastasis.^59^ Increased PD-L1 and CTLA-4 expression are indicative of therapeutic efficacy. In a study of patients with melanoma brain metastases, patients with tumor PD-L1 expression of 5% or more had a higher chance of benefiting from combination therapy (nivolumab and ipilimumab) than those with <5% tumor PD-L1 expression.^60^

Some issues have arisen as a result of the increased use of checkpoint inhibitors. Tumor inflammation and pseudo-progression, which are often seen on imaging, may cause additional symptoms and make tumor growth assessment difficult.^61^ The ability to successfully target brain metastases only among certain patients, such as those expressing high levels of PD-L1, may be a potential limitation of checkpoint inhibitors.^62^ These limitations are significant in some patients to the degree that they may have little to no benefit from the currently available checkpoint inhibition therapies. The efficacy of immune checkpoint inhibitors on brain metastases is dependent on the ability of T cells to become activated by the primary tumor, cross the BBB and/or BTB, and attack tumor cells in the brain.^63^ Most of the data reported for checkpoint inhibitors in brain metastases have combinatorial therapy with other immunotherapeutic agents, radiation, chemotherapeutic agents, or neurosurgery. Additional studies are necessary to explore these challenges and determine how to successfully target tumor cells in the brain.

ACT is a procedure that involves the transfer of autologous immune cells to a recipient to induce an anti-neoplastic effect.^64^ Cells from the primary tumor site or peripheral metastases are cultured in vitro with cytokines and lymphocytes. The immune cells are expanded and re-infused to the patient. One of the most common ACT to treat brain metastases is chimeric antigen receptor T-cell (CAR T-cell) therapy. Chimeric antigen receptors (CARs) are synthetic immune receptors instructing T cells to kill tumors by recognizing unique surface proteins on tumor cells.^65^ The initial CD19 CAR T-cell ACT for metastatic melanoma raised hope for this treatment strategy against brain metastases. A study from 2000 to 2010 identified a subgroup of patients (9.85% of 264 patients) with melanoma brain metastases and treated them with ACT using either autologous tumor-infiltrating lymphocytes or lymphocytes designed to express a T-cell receptor to recognize melanocyte differentiation antigens. Nine of the patients achieved a complete response in the brain and 7 patients reached an overall partial response.^66^

Although ACT for brain tumors is still in the early stages of development and clinical responses are often unsuccessful, these results demonstrate T-cell therapy has potential clinical benefit for patients with brain metastases. Tumor heterogeneity has rendered CAR T-cell treatment for brain and other solid cancers challenging. In fact, this treatment has yet to be proven efficacious in solid tumors. The performance of CD19 CAR T cells emphasizes the importance of a CAR target commonly distributed across tumors. Discovering unique antigens in brain tumors has proven difficult because they express many markers found in normal brain regions (eg, CD133, CD44, Nestin, GFAP) and nonspecific cytotoxic effects in the CNS are much less tolerable than in most other areas of the human body.^65^ Clinical CAR T-cell studies have also reported points of restraint for neurotoxicity and lethal cerebral edema, highlighting the life-threatening risks of immune-inflammatory responses in the CNS.^67^ Almost 12%-32% of patients treated with CAR T cells suffer from extreme neurotoxicity which includes symptoms of confusion, delirium, and seizures. The extent of these neurologic toxicities is referred to as CAR T-related encephalopathy syndrome (CRES). A study by Gust et al reported patients with severe neurotoxicity may have endothelial cell activation which includes intravascular coagulation, capillary leakage, and increased BBB permeability. The cerebrospinal fluid contained high concentrations of inflammatory cytokines leading to pericyte stress, activation of endothelial cells, and further damage to BBB integrity.^67^

Immunotherapy for brain metastases has come a long way since its establishment. The interplay between activating the immune system against tumors while limiting neurotoxic effects is a complex balance. T cells naturally cross the BBB, but the main hurdle with this treatment is determining the proper antigen to activate the cytotoxic T cells. More research is necessary to solidify this as an effective therapy for patients with brain metastases.

## Modulation of BBB Permeability

Bevacizumab is a recombinant humanized monoclonal immunoglobulin G1 antibody that binds to VEGF to decrease endothelial proliferation and formation of new blood vessels.^68^ Bevacizumab in primary brain cancer, such as GBM, is well known to improve progression-free survival when used alone and/or in combination with chemotherapeutic agents.^69^ The objective of bevacizumab is to normalize the vasculature of the tumor and improve oxygenation to aid in delivery of anti-cancer drugs. Bevacizumab also contributes to normalization of the blood vessels in the tumor with low permeability, hence leading to decreased penetration of drugs. Additionally, these tumors would become more invasive by co-opting normal blood vessels.^70^ Preclinical studies suggest long-term use of bevacizumab leads to decrease in BBB permeability.^71^

Recently, bevacizumab has been studied in combination with radiation therapy. The REBECA trial was the first clinical trial to study the effects of bevacizumab and whole-brain radiation therapy in patients with brain metastases. Results demonstrated a synergistic effect between the 2 treatment modalities.^72^ One study combining bevacizumab with stereotactic radiosurgery improved treatment efficacy and reduced edema in a study of patients with lung cancer brain metastases.^73^ Clinical studies in GBM patients show increased progression-free survival in phase II and III trials, but little to no change in overall survival.^74,75^ Bevacizumab is more efficacious as a preventative treatment for brain tissue necrosis than as a tumor treatment with radiotherapy.^76^

Matrix metalloproteinases (MMPs) are zinc-dependent endopeptidases with the primary function to degrade extracellular matrix. These MMPs have role in breast cancer initiation, growth, angiogenesis as well as activation of growth factors. Currently, MMP inhibitors are being studied to evaluate their efficacy in breast cancer. While the use of MMP inhibitors in several brain diseases such as intracerebral hemorrhage, cerebral ischemia, and cold injury has shown to decrease the BBB permeability, there is no concrete evidence to support its efficacy with brain tumors. Studies show MMPs contribute to tumor cells entering and exiting the vasculature to seed in metastatic sites throughout the body. Although results are inconclusive if MMP inhibitors can treat brain metastases, they could potentially prevent metastatic malignant cells invasion.^77^ The role of MMP and its inhibitors may be investigated further in brain metastases.^78^

Aquaporins (AQPs) regulate intra-/extracellular water balance by transportation of fluid across the plasma membranes. Among 13 subtypes of AQP, AQP4 is most abundantly present in the brain and is responsible for cytotoxic edema. Since AQP inhibitors including cryoablation have been in use for the clinical management of breast cancer, inhibition of AQP may be used as an adjunct treatment to lower the BBB permeability.^79^

Dexamethasone is a corticosteroid with anti-inflammatory effects and low mineralocorticoid activity. Among several roles in cancer management, dexamethasone is widely used in controlling pain, nausea, and fatigue. Clinical and preclinical reports suggest dexamethasone can dramatically decrease the BBB/BTB permeability as well as regional vascular tight junction structure.^80^

### Treatments to Increase BBB Permeability

Lack of BBB permeation of therapeutics has fueled research into techniques to increase BBB permeability to increase distribution of drugs into brain and tumor lesions. MRI-guided focused ultrasound is a relatively newer technique for BBB/BTB disruption. The delivery of focused ultrasound at higher energies is able to ablate a tumor mass within the brain (high-intensity focused ultrasound [HIFU]). However, at lower energies in the presence of vascular gas-filled microbubbles (low-intensity focused ultrasound [LIFU]) the LIFU causes a BBB opening.

The LIFU medicated increase in BBB permeability occurs by a combination of physical effects on the NVU and secondary inflammatory responses.^81^ Under the exposure of focused ultrasound, microbubbles undergo oscillations within the vasculature impacting the endothelial cell membrane. The exerted pressure of the microbubble against spaces between endothelia transiently increases the aqueous diffusion of drugs into the brain. The secondary effect of acoustic cavitations includes sterile inflammation. After LIFU, there is a release and elevation of heat-shock protein 70, IL-8, TNF-α, and damage-associated molecular patterns in the parenchyma.^82^ Aravantis et al examined the effect of focused ultrasound with the uptake of 2 relevant chemotherapies, doxorubicin and ado-trastuzumab emtansine^83^ in a HER2 amplified estrogen-dependent model of breast cancer brain metastasis. They observed a 7-fold increase in doxorubicin brain uptake and 2-fold increase in the antibody-drug conjugate. Similarly, trastuzumab plus LIFU increased median survival and reduced tumor volume as compared to non-treated group in a Her2 and neu positive model of brain metastasis of breast cancer.^84^ Despite evidence of preclinical success, LIFU parameters such as including power, cavitation dose, and duration of sonication needs to be elucidated to achieve consistent and reliable BBB/BTB opening in clinical studies.

While radiation remains the standard of care treatment therapy for most brain malignancies, it has been shown that low doses of radiotherapy may enhance BTB permeability to chemotherapy. For example, early work demonstrated that CNS irradiation of 60Gy caused BBB and BTB leakage of horseradish peroxidase, loss of capillary networks, white matter necrosis, and cortical atrophy. These effects were ameliorated 6- to 12-week post-radiation injury.^85^ Later work demonstrated effects were dose-dependent and fractionated doses up to 20-30 Gy increased BBB permeability without producing acute or chronic side effects.

Increased permeability following radiotherapy occurs by a primary physiological effect on the brain endothelium followed by a secondary neuroinflammatory response. The inflammatory response may be through an extracranial abscopal effect where radiation damage at the endothelia causes a release of tumor-associated antigens. Direct effects of radiation include a decrease in tight junction protein expression, decreased endothelial cell density, endothelial apoptosis, and higher transcellular transport. Acute effects post-radiotherapy are initiated by inflammatory mediators like activated astrocytes and microglia, TNFα, IL-6, ICAM-1, and IL-1β.

Reports regarding the extent and time course of radiation-mediated BBB/BTB opening are not consistent. A study by Yuan and colleagues evaluating the effects of fractionated radiotherapy (2Gy, 5 days a week) on brain microvasculature showed BBB permeability did not increase until 90-day post-irradiation.^86^ They found higher vesicular activity, lower tight junction density, and increased number of astrocytes in the brain between 90- and 180-day post-irradiation. However, a separate study found acute increase in BBB permeability 24-48 hours post-radiation after a 20Gy radiation dose.^87^ Interestingly, the radiation-induced increase in microvascular network could be rescued by anti-TNF treatment. A 2016 preclinical study investigated the effects of radiotherapy on tumor burden and permeability in a breast cancer brain metastasis model. The study demonstrated clinically relevant doses of whole-brain radiation of 20Gy fractions reduced tumor volumes of enhancing tumors but not non-enhancing impermeable tumors.^88^

Physical disruption of the BBB can also be carried out by the infusion of a hyperosmotic solution of mannitol (25% w/v) or arabinose into the internal carotid artery. Change in osmolarity of the cerebrovascular endothelial cells causes dilation and shrinkage of the vasculature, leading to an increase in the inter-endothelial space. Widening between the tight junctions (approx. 200 Å) and contraction of endothelial cytoskeleton by calcium causes increase in the BBB permeability, which is highly transient and can last from a few minutes to a few hours.^89^ In addition to higher bulk flow rates, there may also be secondary neuroinflammatory responses with osmotic opening. Higher brain levels of cytokines, tropic factors, damage-associated molecular patterns, and cell adhesion molecules have been observed to occur 5-minute post-infusion. Moreover, sterile inflammatory responses were also observed in the contralateral hemispheres. Neuroinflammatory processes returned back to baseline 96-hour post-osmotic disruption.

While this technique has been extensively explored in primary brain malignancies, its effect on brain metastasis remains unknown. The ability of osmotic disruption to improve delivery of temozolomide was tested in an MGMT-negative lung cancer brain metastasis model. The study revealed BTB disruption enhanced temozolomide delivery within tumors by approximately 3-fold as compared to healthy brain. However, it is important to note BBB disruption with temozolomide was highly toxic and the study group was terminated.

An alternate approach to increase BBB permeability is exploiting the activation of endothelial receptors through natural ligands or their analogs. Biochemical activation of receptors like the adenosine 2A, bradykinin type 2 (B2), calcium-activated potassium channels, or ATP-sensitive potassium channels can increase endocytosis as well as downstream signaling to increase BBB permeation. The effect of bradykinin-induced BBB permeability is dose-dependent, transient, and reversible.^90^ A proposed mechanism for bradykinin-induced BBB breakdown involves increase in trans-endothelial transport by pinocytic vesicles, as animals that were pretreated with imidazole, trifluoperazine or indomethacin had a decreased effect. Endogenous peptides, like bradykinin, increase intracellular cytoplasmic Ca^2+^ levels mediated by endothelial connexin hemichannels. Alternate downstream events include release of free radical oxygen species and arachidonic acid, activation of phospholipase A2, and higher production of IL-1β.^91^

While preclinical studies have promising data, clinical translation has been difficult. First, biochemical modulation of the BBB with bradykinin requires administration of high concentrations of the endogenous ligand, which can cause severe damage to the brain microvasculature. Secondly, bradykinin has a short half-life and very potent metabolites with vasoactive action. This limits the ability of widespread use of bradykinin in the clinic. While selective B2 agonists like labradimil can potentially reduce some the nonspecific side effects, it is yet to be effectively used within the clinic.

In the past decade, laser interstitial thermal therapy (LITT) was developed for treatment of gliomas. Recent evidence suggests this procedure disrupts the BBB.^92^ LITT is a minimally invasive ablative technique that induces cell death of cancerous cells while simultaneously disrupting the BBB for several weeks.^93,94^ The mechanism behind the novel technique is based on the principle cancer cells are more sensitive to thermal damage than healthy cells. The therapeutic window of LITT, however, is small because tumor cells are damaged at 42°C while normal neurons are damaged at 43°C.^92^

### Manipulation of BBB Receptors to Enter the Brain

Drugs or drug delivery systems can be designed to take advantage of the unique BTB in brain metastases. For example, the “trojan horse” method transports drugs across the BBB by attaching an antibody or peptide to a drug/nanoparticle to target receptors along the BBB which facilitate receptor transcytosis. Some of the most common receptors used for this purpose in different brain pathologies are transferrin receptor (TfR), insulin receptor (InsR), and LDL-related protein type 1 (LRP1).^95^

TfRs are expressed on the luminal side of the BBB. The TfR uses receptor-mediated transcytosis to bind transferrin, an iron sequestering peptide, and shuttle iron into the brain. Using this approach, docetaxel-loaded micelles conjugated to transferrin had a 20.8-fold increase in comparison to free docetaxel.^96^ A few in vivo studies have been performed targeting TfR with brain metastasis animal models and show positive results of increased drug uptake in the brain.^97,98^ A study by Wyatt et al assessed the permeability of transferrin-targeted nanoparticles in 3 different models of brain tumors: intracranial, intracardiac, and intravenous (tail vein). They observed different levels of uptake in the models. The intravenous model was the least permeable to their nanoparticles, followed by the intracranial model, with the intracardiac model being the most permeable.^99^ These data highlight the importance of utilizing translationally relevant animal models when evaluating drug delivery to the brain.

A lipid transporter (LRP1 [low-density lipoprotein receptor-related protein 1]) at the BBB binds to LDL and allows lipoproteins to be transcytosed across endothelial cells.^100^ One study utilizing an in vivo model of brain metastases showed upregulation of LRP1 increased transcytosis of nanoparticles loaded with doxorubicin and increased survival of mice bearing brain metastases.^101^ Whereas a preclinical glioma model demonstrated increased brain uptake and survival with angiopep-2 peptide (ligand for LRP1) conjugated to paclitaxel.^102^ More studies are necessary to determine if LRP1 targeted drugs could be efficacious in clinical trials.

Large amino acid transporter 1 (LAT1) transports neutral l-amino acids across the BBB. It is overexpressed in GBM and studies using LAT1 targeting liposomes showed increased brain uptake in glioma models.^103^ One study observed a 60% increase in survival with LAT1 liposomes loaded with a STAT3 inhibitor, WP1066.^104^ The other study noted their LAT1 liposomes loaded with docetaxel were more cytotoxic in the gliomas of their animal model than docetaxel alone.^103^

Another mechanism of manipulating the BBB to deliver drugs to the brain is by inhibition of efflux transporters, which in theory should allow more influx of chemotherapeutic drugs across the BBB and/or BTB. A study in 2019 measured the uptake of radiolabeled erlotinib in the brains of mice after administration of other P-gp/BCRP substrates. There were significant increases in brain uptake of erlotinib, despite complete inhibition of P-gp and BCRP not being achieved. The most promising inhibitor, tariquidar, increased uptake of erlotinib by 69%.^105^ Similarly, animals pretreated with valspodar, a P-gp inhibitor, had increased uptake of paclitaxel by almost 10-fold into the brain and resulted in decreased tumor burden.^106^ Sorafenib, a tyrosine kinase inhibitor, in the presence of elacridar, predominantly a BCRP inhibitor, increased the brain-to-plasma ratio by 5-fold.^107^ Although these inhibitors were effective increasing drug concentrations delivered to the brain, none have significantly increased patient survival in clinical trials.^108^

A clinical study with healthy male patients observed enhanced brain uptake of radiolabeled erlotinib when an oral dose of erlotinib was administered first. The study also investigated the effects of tariquidar administration on erlotinib brain uptake. Tariquidar was not as effective in increasing radiolabeled erlotinib brain concentrations as pre-administration of erlotinib. This is hypothesized to occur due to saturation of the P-gp and BCRP efflux transporters. Although it is important to note the dosage of erlotinib used was much higher than traditionally recommended and has potentially toxic side effects.^109^

## Conclusion

The BTB remains a hurdle in the treatment of CNS tumors. This is notably observed when therapeutics are effective in treating peripheral disease, yet treatment of CNS lesions is largely unsuccessful with the same therapy, presumably because of limited drug penetrance in the central lesion. Currently, in the clinic, mechanisms to alter the BTB of brain metastases specifically and increase drug uptake are unavailable. The typical regimen is to use therapeutics already designed to penetrate the BTB with 60%-80% bioavailability along with use of radiotherapy. Many techniques have been developed to improve drug delivery to the brain. LiFU shows efficacy in increasing BBB permeability and delivering drug, but more studies are needed to determine optimal treatment strategies. Exploitation of transporters at the BBB for drug delivery are promising techniques to increase brain uptake; however, this methodology remains nascent in the clinic.

In the future, it may be beneficial to use BTB molecular differences to target treatment of brain metastases. Ongoing studies of molecular markers of the BTB show differences in pericyte populations, basement membrane formation, and astrocyte attachment to the vessels. One study evaluated vasculature growth patterns of lung, colon, and breast cancer brain metastases from patients. Lung and colon brain metastases had fewer vessels and collagen accumulation in the brain parenchyma, while breast cancer brain metastases had more vessels with collagen accumulation in the tumor core. The vessels also had increased collagen along the walls, increased density and diameter of vessels, added layers of PDGF-β ^+^ pericytes, and detachment of astrocytes.^110^ A preclinical study observed dilated capillaries with increased CD31 expression and desmin^+^ pericytes in a lung cancer brain metastasis model. The study noted a 12-fold decrease in AQP4 along the BTB, which correlates with patient samples.^111^ Additional research is necessary to determine molecular disparities between the BBB and BTB for this to be an effective therapeutic target.

Most preclinical works focused on modulation of the BBB to enhance drug delivery have been done in GBM models. It is important to consider the differences in the permeability of the BTB between GBM and brain metastases when developing treatment strategies. While not perfect, numerous intracranial tumor implantation models can mimic the BTB of central tumors, similarly, intracardiac mouse models produce a BTB that has similar heterogeneity of breakdown as clinical brain metastases. Although some preclinical studies show increased penetrance of drugs, this does not always correlate to decreased tumor burden and increased survival, since concentrations generally are sub-therapeutic. Penetrance, accumulation, and final central lesion concentration of chemotherapeutics are critical for successful clinical trials.
